# How Stock of Origin Affects Performance of Individuals across a Meta-Ecosystem: An Example from Sockeye Salmon

**DOI:** 10.1371/journal.pone.0058584

**Published:** 2013-03-07

**Authors:** Jennifer R. Griffiths, Daniel E. Schindler, Lisa W. Seeb

**Affiliations:** School of Aquatic and Fishery Sciences, University of Washington, Seattle, Washington, United States of America; University of California, Berkeley, United States of America

## Abstract

Connectivity among diverse habitats can buffer populations from adverse environmental conditions, influence the functioning of meta-ecosystems, and ultimately affect the reliability of ecosystem services. This stabilizing effect on populations is proposed to derive from complementarity in growth and survival conditions experienced by individuals in the different habitats that comprise meta-ecosystems. Here we use the fine scale differentiation of salmon populations between diverse lake habitats to assess how rearing habitat and stock of origin affect the body condition of juvenile sockeye salmon. We use genetic markers (single nucleotide polymorphisms) to assign individuals of unknown origin to stock group and in turn characterize ecologically relevant attributes across habitats and stocks. Our analyses show that the body condition of juvenile salmon is related to the productivity of alternative habitats across the watershed, irrespective of their stock of origin. Emigrants and residents with genetic origins in the high productivity lake were also differentiated by their body condition, poor and high respectively. These emigrants represented a substantial proportion of juvenile sockeye salmon rearing in the lower productivity lake habitat. Despite emigrants originating from the more productive lake, they did not differ in body condition from the individuals spawned in the lower productivity, recipient habitat. Genetic tools allowed us to assess the performance of different stocks groups across the diverse habitats comprising their meta-ecosystem. The ability to characterize the ecological consequences of meta-ecosystem connectivity can help develop strategies to protect and restore ecosystems and the services they provide to humans.

## Introduction

There is increasing appreciation for how habitat complexity (including variation in geomorphic, chemical, and thermal properties) can buffer ecosystem function and the reliability of ecosystem services by promoting species and population diversity [1]. Ecosystems filter external climate forces differently such that they may offer higher or lower quality habitat depending on prevailing climate conditions. Over time, habitat conditions may vary inversely with one another producing a temporally variable mosaic of habitat quality on the landscape [2]. The mosaic of habitats on the landscape is not necessarily composed of discrete ecosystems but instead represents a network of heterogeneous habitats that can be conceptualized as a meta-ecosystem with movement of organisms, materials, and energy among component systems [sensu 3]. Biological elements of the ecosystem respond to this heterogeneity, producing spatially variable species or population dynamics [4], [5] and life history diversity [6]. Asynchronous productivity results in more stable aggregated dynamics than that of any individual species or population over time [7] and so too are the derived ecosystem properties [e.g. ecosystem productivity, 8] and services [e.g. fisheries, 9].

The availability of variation in habitat conditions not only facilitates the persistence of distinct populations but can also buffer a single population from environmental variability [10], [11]. For example, butterflies in Britain show more stable population dynamics in landscapes with a broader suite of habitat types and topographic heterogeneity [12]. In order for populations to benefit from habitat heterogeneity, these habitats must be connected such that individuals are able to move among them [13]. Population dependence on different habitats is often associated with migratory species that make feeding, breeding, or overwintering migrations over large distances [described by 14]. Alternatively, connectivity among habitat types at small spatial and temporal scales allows individuals to move in order to negotiate short-term tradeoffs between food quantity and quality, density, optimal environmental conditions, and exposure to predation [15], [16]. Life history diversity within a population can lead to the phenomena of partial migration [reviewed by 17] such that not all individuals move among alternative habitats. The relative proportion of migrants within a population over time may be reflective of the variation in relative habitat quality with higher migration rates associated with greater differences in quality [18] or environmental thresholds [19].

Anadromous Pacific salmon (*Oncorhynchus spp*.) are well known for their large scale migrations between freshwater spawning and rearing habitats and marine feeding habitat. Connectivity between the ocean and freshwater habitat, sometimes thousands of kilometers inland, is necessary for these species to complete their lifecycle. Anthropogenic activities, including dams, irrigation, urbanization, and logging, have threatened connectivity among these ecosystems in many regions [20]. However, during the freshwater rearing stage, connectivity at finer scales is also important for juvenile salmon to negotiate growth and survival trade-offs. In this context, salmon capitalize on heterogeneous habitat within a single lake or river system through population or individual movement. Population movements may indicate a seasonal change in productivity among habitats such as offshore movement by juvenile sockeye salmon [*O. nerka*, 21] or a balance between feeding opportunity, thermal conditions, and predator avoidance [e.g. diel vertical migration, 22,23]. Moreover, alternative movement strategies in salmon populations are common, with some individuals in the population occupying a single habitat during a life-stage while other individuals move among alternative habitats in response to habitat quality [24]. Habitats may offer tradeoffs between high resource quality and profitable abiotic conditions (e.g., temperature). Coho salmon (*O. kisutch*), for example, that forage in cold, food rich habitats but move to warm, food poor habitats to process food grow faster than individuals that do not move among habitats [25].

During their freshwater life history phase, juvenile salmon can not only exploit heterogeneous habitat within a single lake or stream but can move throughout watersheds. Some coho salmon exhibit an alternative strategy in which individuals migrate downstream into estuaries in their first year of life and then return upstream to overwinter in freshwater [26]. Similarly, juvenile steelhead (*O. mykiss*) have been shown to exploit estuarine connectivity during freshwater rearing without continued migration to the ocean within the same year [27]. Sockeye salmon also exhibit inter-lake migrations from high to low density lakes [28] or among lakes with very different abiotic conditions [29].

The attributes of movers in salmonid populations and the ultimate consequences for those individuals and their populations are context dependent. A variety of factors may influence an individual’s propensity to migrate including competition, food availability, and population density [17] which may be reflected in their physical characteristics such as size or body condition. In some systems it appears that while movers and residents do not exhibit initial differences in physical condition, movers have higher growth rates upon moving to alternative habitats [24]. In other systems, individuals that become emigrants may be of lower or higher condition than residents depending on the environmental conditions in a given year [29].

Assessing the success of movers versus residents poses a challenge when individuals of one population immigrate into new habitats that are already occupied by a different population of the same species. This is likely to happen when migrants exploit habitat connectivity at the watershed scale. Furthermore, movers could have direct or indirect effects on the resident individuals in their new habitat. Evaluating the success of alternative movement strategies as well as the interactive effects among populations requires the identification of individuals to their population of origin. Population structure is often cryptic and only detectable with genetics or intensive tagging studies [30]. Genetic tools can provide a useful and less time-consuming alternative to tagging studies, particularly for large systems with high organism densities where recapture rates are low. Genetic techniques are particularly well-developed for Pacific salmon [31], [32] due to substantial interest in population-level management at both the state and federal level. Furthermore, because of strong natal homing by spawning adults [33], salmon populations are highly differentiated at relatively fine spatial scales [34], [35], [36]. Specifically, single nucleotide polymorphisms (SNPs) have become a common and robust tool to allocate Pacific salmon of unknown origin to known spawning populations [37], [38].

The Chignik watershed on the Alaska Peninsula provides the opportunity to investigate individual performance among alternative rearing strategies in a sockeye salmon meta-ecosystem. Freshwater life histories of sockeye salmon have historically differed between natal lakes in this watershed. Juveniles from Black Lake (upper watershed) spend one year in freshwater and individuals from Chignik Lake (lower watershed) spend two years in freshwater, reflecting the thermal conditions and relative productivity between the two lakes [39]. Downstream emigrations by a proportion of the Black Lake juvenile sockeye salmon population to Chignik Lake appear to be common in this meta-ecosystem, however [29], [39], [40]. Mid-summer juvenile emigrations are only in the downstream direction, and Black Lake juvenile sockeye salmon emigrants spend the remaining portion of their freshwater residence in non-natal habitat [29]. In recent decades, median emigration dates ranged from mid-June to mid-July with the majority of the emigration concluded by the end of July [29]. Furthermore, downstream emigrants (captured downstream of the lake outlet) have a lower body condition than fish that remain in Black Lake throughout the summer [29].

Once these emigrants enter Chignik Lake, however, their performance in non-natal habitat is unknown. Furthermore, because fish sampled in Chignik Lake cannot be visually identified to stock, characterizing the body condition and growth of Chignik Lake stocks has been historically limited to scale pattern analysis which made assumptions about how growth differed between stocks. Recently, SNPs have been used in the Chignik watershed to assess stock specific characteristics in a common rearing environment during a single summer [2008, 41]. Simmons et al. [41] found that a substantial fraction (33%) of the juvenile sockeye salmon rearing in Chignik Lake in mid-July were of Black Lake origin, which increased to 46% at the end of August. Simmons et al. [41] were able to compare the performance of individuals among habitats for a subset of the individuals sampled, but 45 SNP markers were only able to robustly assign 40% of the individuals captured.

Here we build upon the work of previous studies and use the fine scale differentiation of salmon populations among diverse lake habitats on the Alaska Peninsula to assess how rearing habitat and stock of origin affect the body condition of juvenile sockeye salmon. We were able to robustly assign individuals of unknown origin to stock groups using a greater number of SNPs than previously available and in turn characterize ecologically relevant attributes across habitats and stocks. We addressed the following questions. 1) How variable is the stock composition of juvenile sockeye salmon in a common rearing environment (Chignik Lake) among years? 2) Does habitat quality differ among lakes as expressed by juvenile sockeye salmon body condition? 3) Is emigration from warm (Black Lake) to cold (Chignik Lake) summer habitat linked to body condition? 4) In a shared rearing environmental, what are the relative body conditions of natal (Chignik Lake) versus non-natal (Black Lake) individuals?

## Methods

### Ethics Statement

Sample collections and methods were permitted under Alaska Department of Fish and Game (ADFG) permits SF2010-094 and SF2011-121. All protocols complied with the University of Washington IACUC permit 3142-01.

### Study Site

**Figure 1:**
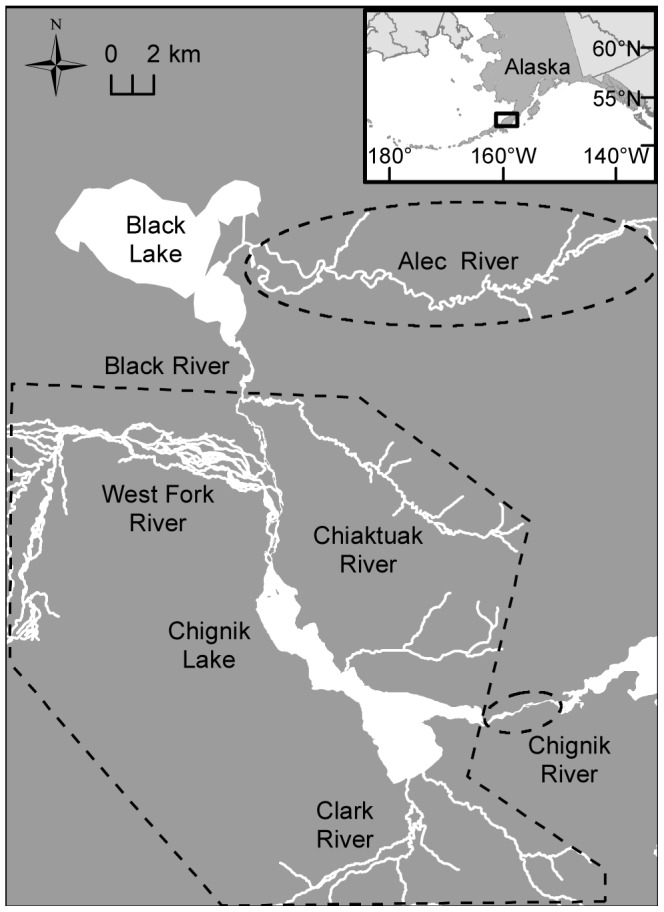
Map of the Chignik watershed. The spawning habitat encompassed within each stock group is outline by a dashed polygon. Black River spawning populations occur primarily in the Alec River and adjacent Fan Creek (not shown). Chignik Lake spawning populations include the West Fork and Chiaktuak Rivers, Chignik Lake beaches, Clark River and other minor tributaries. The Chignik River spawning population occurs downstream of the Chignik Lake outlet.

In the Chignik watershed, Alaska Peninsula, USA (Figure 1), sockeye salmon (*O. nerka*) are the numerically dominant anadromous species and support a valuable commercial fishery (average annual harvest 1.7 million since 1977, data from ADFG) and a local subsistence harvest. Sockeye salmon spawn in tributaries to both Black and Chignik lakes, rear in freshwater for 1–2 years, migrate to the ocean for 3 years on average, and then return to natal streams and lake beaches to spawn. The number of spawners (escapement) is tightly controlled by ADFG and was relatively constant during our study years. The escapement for the Black Lake stock was 391,474 in 2009 and 432,535 in 2010. In Chignik Lake, juvenile sockeye captured may be age-0 or age-1. Escapements producing the juvenile sockeye we sampled were 328,479 (2008), 328,586 (2009), and 310,634 (2010).

The attributes of rearing habitat for juvenile sockeye salmon in the Chignik watershed are diverse. Shallow Black Lake (4 m max. depth) is a warm, turbid, and productive lake in the upper watershed. Black Lake is also experiencing geomorphic evolution on ecological time scales and has lost ∼40% of its volume since 1960 [42]. In contrast, deep and cold Chignik Lake (60 m max. depth) downstream has maintained a stable volume over recent decades. Differences in sensitivity to air temperature reflect the geomorphic differences between the lakes. In our sample years, mean daily July and August surface water temperatures in Chignik Lake were 10.8°C (2010) and 10.7°C (2011) while in Black Lake they were 13.1°C (2010) and 12.6°C (2011). Furthermore, air temperatures have increased 1.4°C on average in the watershed between 1960 and 2005 [43].

### Sample Collection 2010–2011

Juvenile sockeye salmon in Chignik and Black lakes were sampled at the end of August using townets. In 2010, sample dates were August 25^th^ and August 28^th^ in Chignik Lake and Black Lake, respectively. In 2011, samples were collected on August 24^th^ in Chignik Lake and August 25^th^ in Black Lake. Five sites on Chignik Lake were sampled using a 2 m×2 m net, which was pulled at the lake surface between two boats for duration of 10 minutes per set. The same protocol was used to sample five Black Lake sites but a 1.2 m×1.2 m net was deployed. If samples were large, a known fraction of the catch was retained. Fish were euthanized in a buffered MS222 solution and were returned to the lab for processing. Sockeye salmon were measured to the nearest mm (fork length) and weighed to the 0.1 g. Genetic samples were collected from Chignik Lake by removing the entire caudal fin. Sample tissues were pressed to gridded filter paper and air dried for later DNA extraction. The association between each fish’s length, weight, and genetics sample was retained. Data deposited in the Dryad repository: http://dx.doi.org/0.5061/dryad.jn14d.

### Laboratory Analysis

A subset of individuals captured in Chignik Lake was genotyped in 2010, and all captured individuals in 2011 were genotyped. In 2010, samples were grouped by lake section, north (2 sites) and south (3 sites), and 285 samples were selected from each. The majority of fish captured were between 61–70 mm in the north and 61–75 mm in the south. Because we believed that length may reflect stock at the tails of the distribution, the samples from all fish ≤60 mm and >70 mm were taken for analysis (n = 98) for the northern section. The remaining 187 samples were taken in random draws in proportion to the sample numbers in the remaining two 5-mm length bins. Similarly, in the south area, samples from all fish ≤60 mm and >75 mm were retained for analysis (n = 48). The remaining 237 samples were taken in random draws in proportion to the sample numbers in the remaining three 5-mm length bins.

Genomic DNA was extracted following standard protocol with Qiagen DNeasy 96 Tissue Kits. Multiplex preamplification PCR was conducted to reduce error and failure rates in case of low concentrations of template DNA [44]. A 96 SNP panel was assayed using TaqMan reactions as in [45]. The 96 SNP panel included 3 mitochondrial SNPs and 93 nuclear SNPs now used in mixed stock analyses by ADFG [46]. The Fluidigm Biomark 96.96 was used to genotype the samples. For quality control, 8 out of every 95 individuals were reanalyzed to confirm that genotypes were reproducible and identify laboratory errors.

### Genetic Analysis

ADFG provided the genotypes for the Chignik watershed baseline populations [46]. Monomorphic loci were identified and removed prior to further analyses. We followed the approach of Creelman et al. [36] for dealing with loci in linkage disequilibrium (LD). *Tf_ex11-750* was dropped from the LD pair *Tf_ex11-750* and *Tf_in3-182*. In case of the two MHC loci (*MHC2_190* and *MHC2_251*), we treated them as phenotypic characters [47] to retain the information contained by both loci. The three mitochondrial loci were combined into a composite haplotype.

All stock identification analyses were carried out using a Bayesian approach developed by Pella and Masuda [48] (BAYES). Baseline populations were pooled to 13 populations following Creelman et al. [36]. Five populations belonged to the Black Lake stock group, seven to the Chignik Lake stock group, and one to the Chignik River stock group (geographic extent shown in Figure 1). A uniform prior was used with each pooled baseline population given equal weight and the probabilities summing to one. For each mixture, 3 Monte Carlo Markov chains were run with randomized starting locations. Each chain had a length of 140,000 iterations with every 7^th^ sample retained for a total of 20,000 samples per chain (burn-in 10,000). This level of thinning was determined by the Rafterty-Lewis diagnostic [49] across multiple runs. A unique combination of starting stock proportions was used for each chain. Starting proportions of 0.3 were randomly assigned to 3 populations and the remaining 0.1 divided among all other populations.

### Mixture Allocations

The relative contribution of each stock group to the unknown mixture sample was assessed using mixture allocation. BAYES established posterior densities of mixture proportions at the stock group level (Black Lake, Chignik Lake, Chignik River) for each chain. Convergence of the posterior densities among the chains was verified using the Gelman-Rubin diagnostic [50] and visual assessment. A 95% credibility interval, mean, and median stock group proportions were calculated for the combined chains for each mixture. In 2010, we genotyped all fish in the tails of the length distribution to ensure individual performance was well characterized across the length distribution. To avoid bias in our mixture allocation analysis, we used only the 61–75 mm fish (center of the distribution) randomly selected for genotyping from the south section of the lake (n = 237) in 2010. The majority of fish in 2010 (94.4%) were captured in the south section of the lake and 92% of these fish were from 61–75 mm in length. Therefore, we believe the stock composition of this random sample best reflects the lake wide composition.

### Individual Assignment

The ability to robustly assign individuals to a stock group depends on the number of markers and the level of differentiation among reporting groups [51]. Our ability to assign individuals in the Chignik watershed has increased since past studies have been conducted due to the increase in the number of SNP markers available (96 rather than 45 as in Simmons et al. [41]).

We assessed the individual assignment ability of the baseline by conducting tests using mixtures created from individuals from known baseline populations following the methods of Simmons et al. [41]. We randomly selected individuals from the baseline populations to create a test mixture of 200 individuals and generated a new baseline without the selected individuals. The representation of each stock group in the mixture reflected observed mixture allocations to stock group in 2010 and 2011 (25% Black Lake, 75% Chignik Lake). We repeated the randomization process 10 times each time generating test mixtures and baselines with the same stock group proportions. We then used BAYES to assign posterior densities of mixture proportions to stock groups (as above) as well as assign individuals to the 13 populations. For each individual in a test mixture, we summed the population level assignments by stock group. We then assessed the number of individuals assigned to each stock group at assignment thresholds ranging from 50 to 90% [41]. At each threshold level, we calculated the error rate by determining the proportion of individuals incorrectly assigned to that stock group. We calculated the mean error rate and standard deviation across all ten test mixtures by threshold and stock group. To determine the threshold to use for further analyses we sought to maximize the number of individuals assigned while minimizing the rate of incorrect assignment.

Assignment of unknown individuals to stock group in 2010 and 2011 was conducted using BAYES as previously discussed. We used the 80% threshold to assign individuals to a reporting group based upon the analyses above. This allowed the use of individual attributes (length, weight, condition) of each fish to define the attributes of each stock group by rearing environment and movement status.

### Stock of Origin, Rearing Lake, and Body Condition

For individuals assigned to either the Chignik Lake or Black Lake stock as described above, we tested for differences in length between three combinations of stock of origin and location of capture: natal rearing environment of different stocks (Black Lake residents and Chignik Lake residents); emigrant/resident status of the same stock (Black Lake emigrants and Black Lake residents); and common rearing environment but different stocks (Black Lake emigrants and Chignik Lake residents). Within the comparison between Black Lake emigrants and Chignik Lake residents, there was a third group, which were the individuals not assigned at the 80% threshold. Given highly unequal sample sizes for most of the comparisons, we first tested for homogeneity of variances using Bartlett’s test [52]. If variances were homoscedastic, we used Analysis of Variance (ANOVA) while if they were heteroscedastic we used the non-parametric Kruskal-Wallis test [52].

To explore the differences in the length-mass relationship and the relative body condition of the above pairs, we assessed four alternative regression models to predict fish mass. This approach is consistent with previous work in the watershed [41] and was suggested by Cone [53] as the preferred way to evaluate fish condition. In the first model to compare stocks rearing in their natal lake (Black Lake residents and Chignik Lake residents), all individuals (*j*) belonging to the stock groups (*i*) shared a slope and an intercept relating mass to length. The second model had different intercepts by stock group but the same slope while the third model had the same intercept but different slopes. The final model had different intercepts and slopes for each stock group.
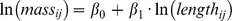


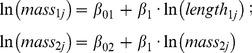


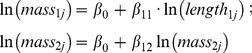


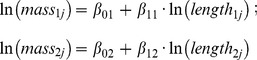



The Black Lake emigrant versus resident comparison (Black Lake emigrants and Black Lake residents) used the same model approach where individuals were grouped by location of capture instead of stock group. Finally, the shared rearing environment comparison (Black Lake emigrants and Chignik Lake residents) were compared using the same model framework. In these two comparisons samples sizes were unequal because of the few Black Lake origin individuals identified in Chignik Lake.

Models were compared using Akaike Information Criteria for small sample sizes (AICc) [54]. Additionally AIC weights (*w_i_*) [54] were calculated for each model within a comparison. Given the suite of models considered, each *w_i_* is the estimated probability that the given model is the best model for the data.

These analyses included fish that were individually assigned to a reporting group at the 80% level. To test the robustness of our results to the assignment threshold used, we compared our results to those obtained when using a 70% (less conservative) or 90% (more conservative threshold (Table S1, Figure S1, Figure S2, and Figure S3). Analyses were conducted using R statistical software [55] including the package “AICcmodavg” [56].

## Results

### Sample Collection 2010–2011

In Chignik Lake, 1000 juvenile sockeye salmon were sampled for length, mass, and fin clip in 2010 and then later sub-sampled for genotyping. In 2011, catch rates were lower, and all sockeye salmon caught at all sites were retained for later analysis (n = 233). In Black Lake, juvenile sockeye sample sizes were 341 and 770 in 2010 and 2011, respectively.

### Laboratory & Genetic Analysis

Five hundred-seventy fish were genotyped from 2010 samples and 233 fish were genotyped from 2011 samples. The assay for the *SUMO1-6* locus failed for all samples and was excluded from the analysis. In 2011 the locus *U1016-115* was also excluded due to assay failure. Two loci were monomorphic (*metA-253, txnip-401*) across the Chignik populations and were not used in further analyses. Additionally, in 2010 two fish were missing genotypes for at least 15% of the loci and were excluded.

### Mixture Allocations

**Figure 2:**
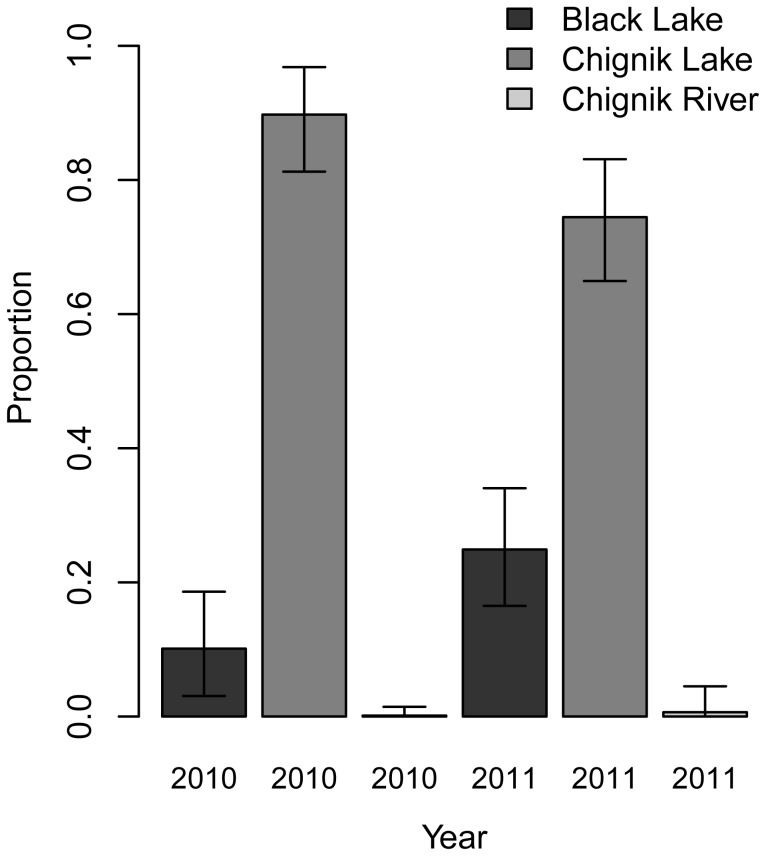
Stock group mixture proportions in Chignik Lake in August. The 2010 and 2011 mixture allocation was based on the 96-SNP baseline [46]. The 2010 mixture composition is based upon the randomly sampled individuals from the south section of the lake that reflected the majority of the catch in 2010 (see Methods for additional details).

Here we report the mean of the posterior density distribution for each stock group and the 95% credibility interval. In 2010, Chignik Lake August stock composition (n = 236, estimated only from the randomly selected individuals in the south lake section) was 10.1% (3.1–18.6) from Black Lake emigrants, 89.7% (81.2–96.8) from Chignik Lake residents, and 0.1% (0–1.45) from Chignik River (Figure 2). In 2011, Chignik Lake August stock composition (n = 233) was 24.9% (16.5–34.1) from Black Lake emigrants, 74.5% (64.9–83.1) from Chignik Lake residents, and 0% (0-.05) from Chignik River (Figure 2).

### Individual Assignment

Individual assignment of mixtures comprised of known individuals demonstrated that the 80% threshold assigned a substantially larger number of individuals than the 90% level and still retained low error. At the 80% threshold, on average 75% of the individuals in the mixture were assigned to either Black Lake or Chignik Lake stock groups. The mean error rate for individuals assigned to Black Lake was 11% (SD ±7%) while the Chignik Lake error rate was 4% (SD ±2%). At the 90% threshold, 59% of individuals were successfully assigned to a stock of origin and there was a greater decrease in the proportion of fish assigned to Black Lake as opposed to Chignik Lake. Mean error rates at the 90% threshold were 3% (±4%) for Black Lake and 3% (±2%) for Chignik Lake.

Overall, we were able to assign 78% and 80% of individuals to a stock group at the 80% threshold for 2010 and 2011, respectively (Table 1). The majority of individually assigned fish were from the Chignik Lake stock due to their numerical dominance in the mixtures in both years. Of the 568 individuals sampled in 2010, 34 were assigned to Black Lake and 416 were assigned to Chignik Lake. In 2011, 31 of 233 individuals were assigned to Black Lake and 150 to Chignik Lake.

**Table 1:** The proportion of the sample in each year assigned to each stock group at the 80% probability threshold.

Year	Black Lake	Chignik Lake	Chignik River	Not Assigned
2010	0.06	0.73	0.00	0.20
2011	0.13	0.64	0.00	0.22

### Stock of Origin, Rearing Lake, and Body Condition

We used the individual assignments at the 80% threshold to assess the length distributions and relative body condition of juvenile sockeye salmon among stocks and rearing lakes. Our analyses show that lake rearing habitat strongly affects juvenile sockeye salmon body condition. Differences in body condition differentiated emigrant (low condition) versus resident (high condition) individuals within a single stock group (i.e., from Black Lake). Despite emigrants originating from the more productive lake, they did not differ in body condition from the individuals originating the lower productivity, recipient habitat. While populations exploit diverse habitats, these habitats differ in productivity, and emigration may not improve attributes such as body condition.

### Comparing Two Natal Lakes: Black Lake Residents Versus Chignik Lake Residents

In both years, there were significant differences in length between stocks rearing in their natal lakes (2010: df = 1, K-W χ^2^ = 89.4743, p<0.001 2.2×10^−16^; 2011: df = 1, K-W χ^2^ = 134.0431, p<0.001). In 2010, Black Lake residents were longer (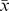 = 69.7 mm, sd = 5.4) than Chignik Lake residents (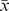 = 65.1 mm, sd = 7.9), however the reverse was true in 2011 (Black Lake residents: 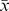 = 64.0 mm, sd = 5.4; Chignik Lake residents: 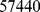 = 70.5 mm, sd = 8.2).

**Figure 3:**
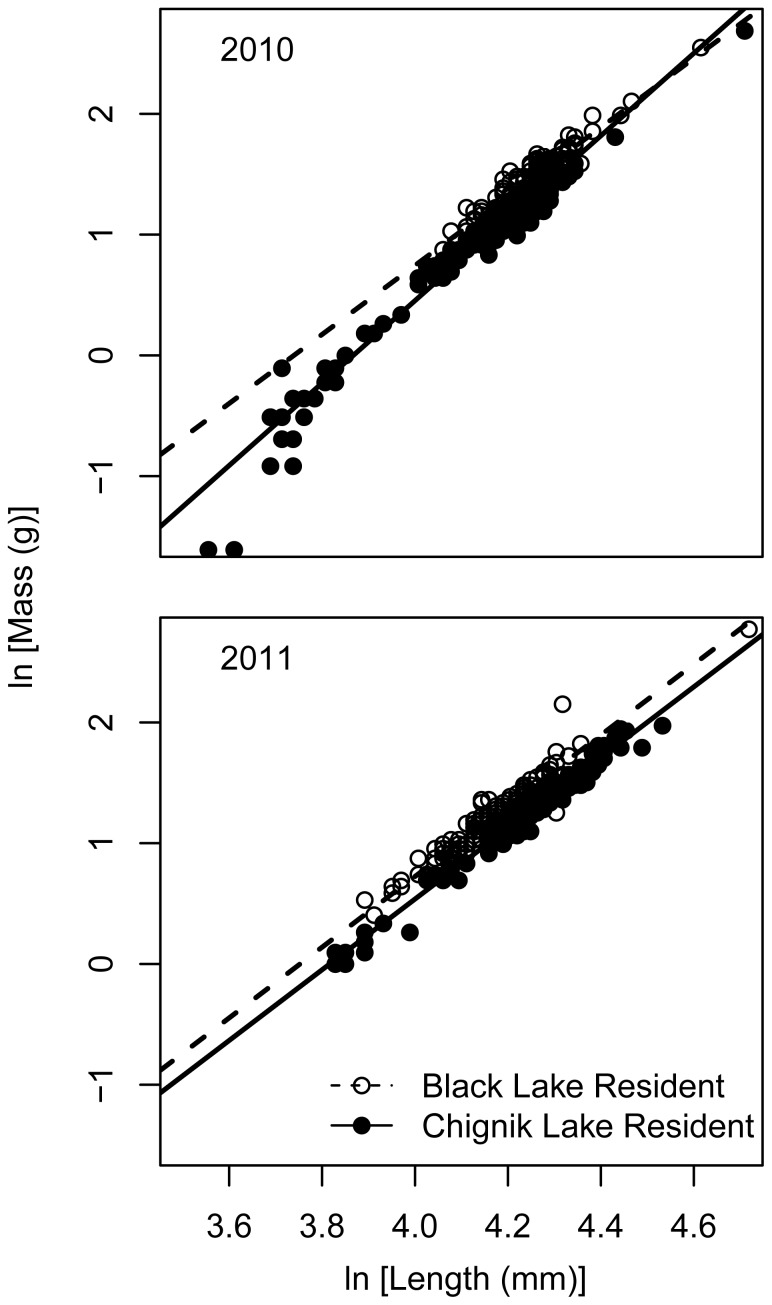
Length-mass relationship for sockeye salmon rearing in their natal lake of origin. These populations are referred to in the text as Black Lake residents and Chignik Lake residents. Circles are individual fish and the lines represent the AICc selected model. For 2010 the best model included different intercepts and slopes by natal lake of origin and capture. For 2011 while the best model included different intercepts and the same slope by natal lake of origin and capture, two other models were within 2 AICc units (Table 2).

There were clear differences in body condition among individuals rearing in their natal lakes. Black Lake residents were of higher body condition in both 2010 and 2011 than Chignik Lake residents (Figure 3). In 2010, there was strong support for the different slope and intercept model (w_i_ = 1.00). This is probably because the Black Lake residents had a much narrower length range than Chignik Lake residents and small Chignik Lake residents had very low body condition. In 2011, the support was strongest for a different intercept and same slope model, but there was also similar support for models with either different slopes or different intercepts (Table 2).

**Table 2:** Comparison of alternative condition factor models**.**

Model	2010		2011	
	ΔAICc	w_i_	ΔAICc	w_i_
**Black L. residents & Chignik L. residents**				
B_0_; B_1_	278.86	0.00	249.35	0.00
B_01_ & B_02_; B_1_	26.49	0.00	0.00	0.49
B_0_; B_11_ & B_12_	30.29	0.00	0.98	0.30
B_01_ & B_02_; B_11_ & B_12_	0.00	1.00	1.68	0.21
**Black L. residents & Black L. emigrants**				
B_0_; B_1_	72.30	0.00	85.92	0.00
B_01_ & B_02_; B_1_	3.05	0.16	0.78	0.28
B_0_; B_11_ & B_12_	4.44	0.08	0.00	0.41
B_01_ & B_02_; B_11_ & B_12_	0.00	0.75	0.56	0.31
**Black L. emigrants & Chignik L. residents**				
B_0_; B_1_	7.96	0.02	0.93	0.30
B_01_ & B_02_; B_1_	4.71	0.08	2.91	0.11
B_0_; B_11_ & B_12_	5.15	0.06	2.95	0.11
B_01_ & B_02_; B_11_ & B_12_	0.00	0.84	0.00	0.48

Models include: the same intercept and slope for each factor (B_0_, B_1_); different intercepts and the same slope by factor (B_01_ & B_02_, B_1_); same intercept but different slopes by factor (B_0_; B_11_ & B_12_); and a different intercept and slope by factor (B_01_ & B_02_; B_11_ & B_12_). The ΔAICc value and AICc model weight (*w_i_*) are given for each model.

### Home Versus Away: Black Lake Residents Versus Emigrants

In 2010 there was a significant difference in length between Black Lake emigrants and residents (df = 1, Kruskal-Wallis (K-W) χ^2^ = 12.0891, p = 0.005) in which Black Lake residents were longer than individuals that had immigrated to Chignik Lake (Black Lake emigrants; 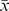 = 64.2 mm, sd = 9.5). No difference in length was detected among emigrants and residents in 2011 (Black Lake emigrants; 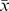 = 65.2 mm, sd = 7.7).

**Figure 4:**
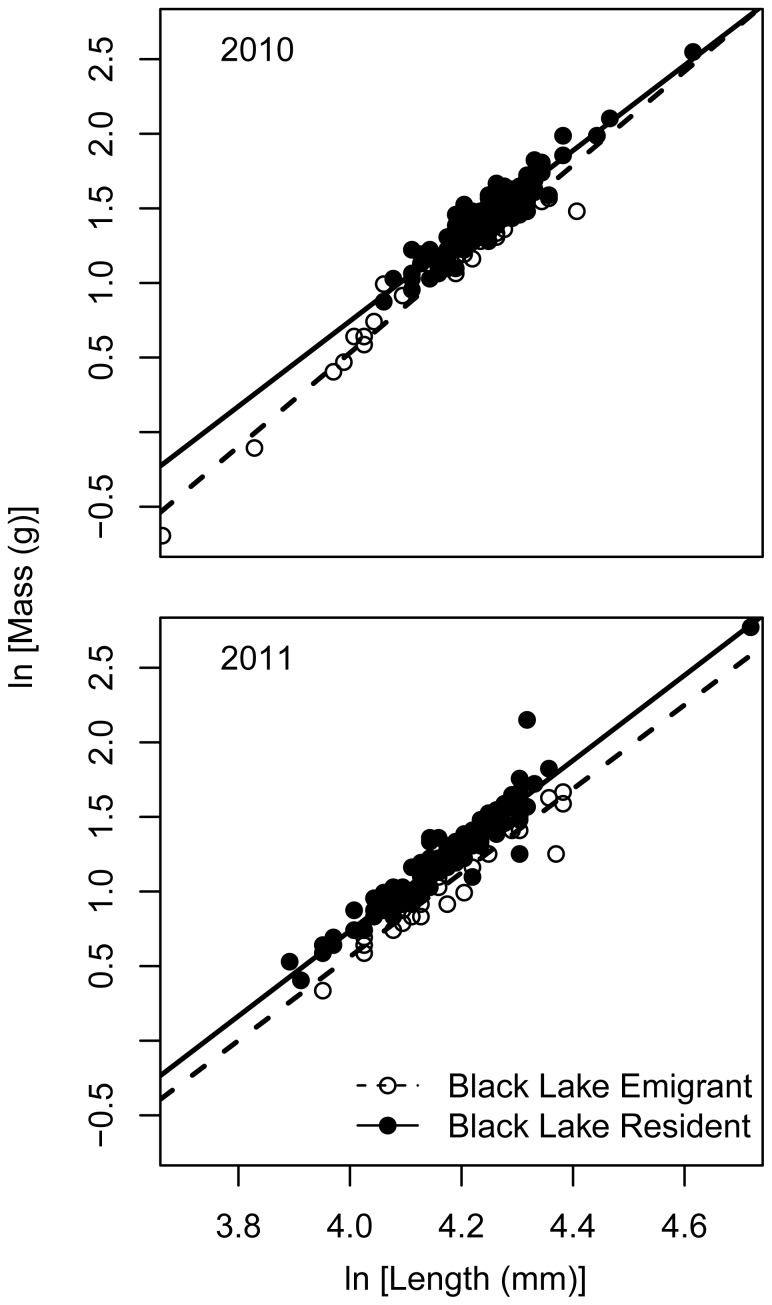
Length-mass relationship for sockeye salmon of Black Lake origin by lake of capture. These population sub-groupings are referred to in the text as Black Lake emigrants and Black Lake residents. Circles are individual fish and the lines represent the AICc selected model. For 2010, the best fit model contained different intercepts and slopes by lake of capture. For 2011, the model shown has the same intercept but different slopes by lake of capture. This model had only slightly greater support than two other models (see Table 2).

In both 2010 and 2011, Black Lake emigrants were of lower body condition than Black Lake residents (Figure 4). In 2010, there was strong model selection support for a model with different intercepts and slopes (w_i_ = 0.75) likely driven by the low body condition of smaller Black Lake emigrants. In 2011, there was no support for the null model but relatively similar support for the other three models (Table 2).

### Locals Versus Migrants: Chignik Lake Residents Versus Black Lake Emigrants

We found significant differences in 2011 among-group lengths (ANOVA, df = 230, F = 7.1867, p = 0.001) but not in 2010 (ANOVA, df = 565, F = 0.4104, p = 0.66331) (mean lengths provided in above sections). A Tukey test for multiple comparisons indicated that in 2011 Black Lake emigrants were significantly smaller than Chignik Lake residents (p = 0.004) but there were not significant differences between either group of known origin and unassigned individuals captured in Chignik Lake.

**Figure 5:**
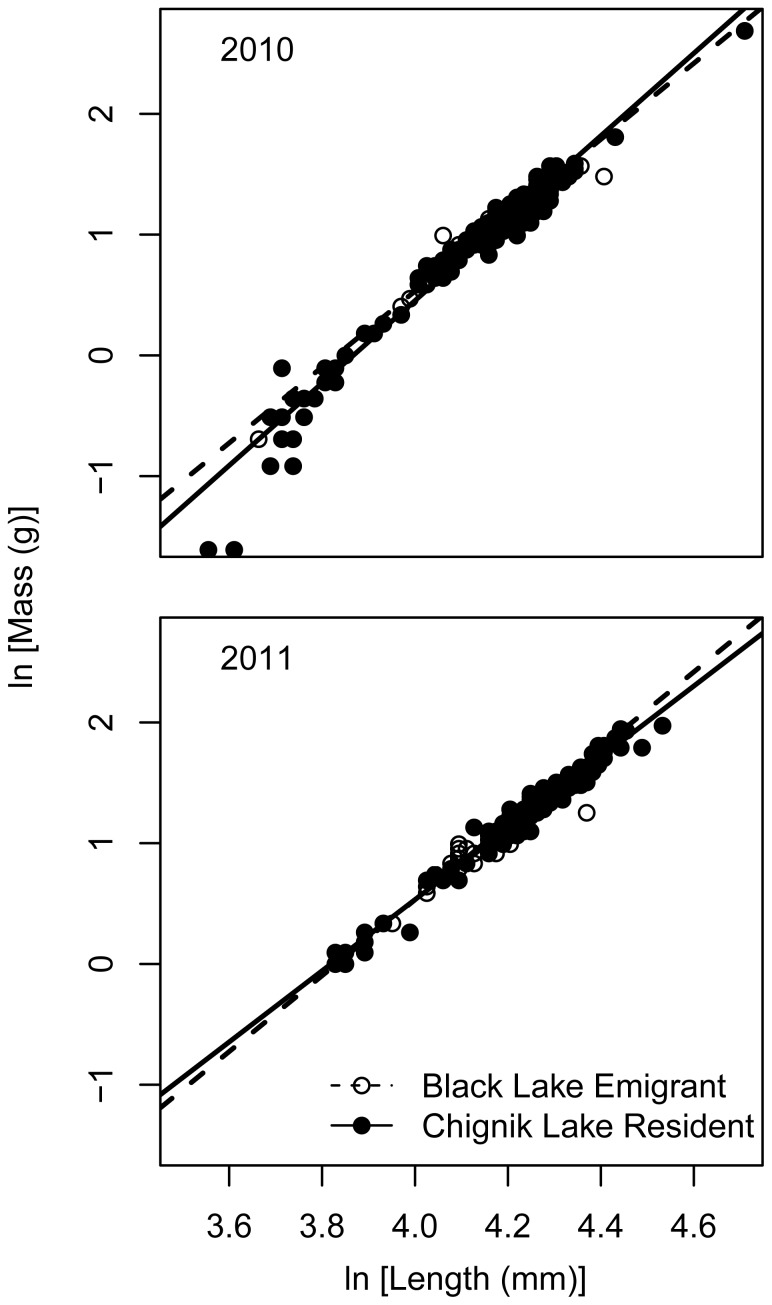
Length-mass relationship for sockeye salmon captured in Chignik Lake by natal origin. These populations are referred to in the text as Black Lake emigrants and Chignik Lake residents. Circles are individual fish and the lines represent the AICc selected model. For 2010 and 2011, the best fit model contained different intercepts and slopes by natal origin. However, for 2011 this model differed less than 2 AICc units from a model with the same slope and intercept for both stocks (Table 2).

In 2010 there was strong support for a model describing the relationship between length and mass with different slopes and intercepts by natal origin (Table 2). Small Black Lake emigrants had a higher body condition than small Chignik Lake residents (Figure 5)_._ As length increased, however, Chignik Lake residents increased in mass more rapidly than Black Lake emigrants. In 2011, there was little visual difference between stocks in their body condition and no model showed substantially stronger support than the shared slope and intercept model (Table 2).

## Discussion

Our mixture analyses showed that juvenile sockeye salmon spawned in Black Lake tributaries made up a substantial but variable proportion of the fish that were rearing in Chignik Lake by the end of the growing season when compared to a survey from 2008 [41]. Using individual genetic assignment to stock of origin, we characterized the body condition of juvenile sockeye salmon residents in their natal lakes as well as those that immigrated to new habitat. Individuals from Black Lake that were rearing in their natal habitat were in substantially better body condition than Chignik Lake fish rearing in their natal, less productive habitat. Juvenile sockeye salmon that emigrated from Black Lake to Chignik Lake tended to have lower body condition near the end of their first growing season than individuals that stayed in their natal Black Lake habitat. Finally, within the common rearing environment of Chignik Lake, fish of Black Lake and Chignik Lake origin had similar body conditions, and the subtle differences detected were size-dependent in the year they were statistically significant.

Residency in productive, warm Black Lake led to highest body condition for juvenile sockeye salmon observed throughout the Chignik watershed. This result likely reflects the differences in ecosystem productivity between Black Lake and Chignik Lake. Further, high body condition of fish rearing in Black Lake may indicate that successful Black Lake residents are able to achieve critical length thresholds earlier in the season and switch to an energy allocation strategy that favors overwinter survival by allocating more energy to storage rather than further growth in length [57]. Mean length comparisons between Black Lake residents and Chignik Lake residents produced opposite patterns in 2010 and 2011. We think this is likely caused by changes in the Chignik Lake age composition (relative proportions of age-0 and age-1) rather than by differences in lake productivity among years. Age composition data were not collected, however.

Poorer body condition emigrants from Black Lake were always present in Chignik Lake but made up a variable proportion of the juvenile sockeye salmon. While credibility intervals show a slight overlap between 2010 (Black = 3.1–18.6%) and 2011 (Black = 16.5–34.1%) these proportions are quite different from those observed in August 2008 (Black = 37–56%). Westley et al. [29] showed that Black Lake emigrants were of lower body condition when departing Black Lake in early to mid-summer than Black Lake residents. We show that these individuals continue to have lower body conditions in alternative rearing habitat. Given the emigration timing reported for recent decades [29] as well as the substantial fraction of emigrants observed in Chignik Lake in July by Simmons et al. [41], we believe that emigrants have likely spent a month rearing in Chignik Lake and that their body condition is reflective of Chignik Lake growth conditions. Their convergence on Chignik Lake growth potential is also reflected in the shared body condition with Chignik Lake residents in the common rearing environment in 2010 and 2011 [consistent with 41].

Interestingly, while earlier observations of poor condition Black Lake emigrants occurred during the extremely warm summers of 2005 and 2006 [29], we show that this also occurs during more average climate conditions. Mean Black Lake temperature from June 12 – August 26 was 12.6°C and 12.1°C in 2010 and 2011, respectively, and the maximum temperature was 15°C. These temperatures were substantially cooler than when poor body condition emigrants were observed in 2005 and 2006. In those years, the mean water temperatures over the same period were 14.1°C and 12.4°C with maximum temperatures reaching over 17°C in both years. If sockeye salmon are feeding at maximum consumption, the optimal temperature for growth is 15°C [58], however, if food is limited optimal growth temperatures are lower. Therefore, the coolest temperatures of the last decade may be at optimal growing conditions in Black Lake while the warmest years are likely sub-optimal for much of the population.

However, our results indicate that conditions are limiting for growth in Black Lake for at least a fraction of the population in Black Lake even during cool summers. For these individuals, emigrating downstream may offer benefits even though growth potential in Chignik Lake is lower. These cooler temperatures in Chignik Lake, although reducing the scope for growth, may also reduce metabolic stress and potentially improve survival. A longer growing period in fall due to Chignik Lake’s large thermal mass may also provide growth opportunities unavailable in Black Lake in the fall. Finally, it is unclear whether Black Lake emigrants ultimately show differences in freshwater rearing duration. Given little differences in length with Chignik Lake individuals (some of which are age-1), Black Lake emigrants may achieve sufficient length to smolt in the following spring or they may rear an additional year in freshwater. The relationship between condition of downstream emigrants and the duration of freshwater rearing could be important for quantifying the importance of emigration for survival. Based on ADFG brood tables, however, there appears to be no large scale shifts in Black Lake freshwater age composition seen in returning adult sockeye between 1922 and 2010 (ADFG, unpublished).

The proportion of juvenile sockeye of Black Lake origin in Chignik Lake is a function of both the downstream emigration rate and the production of sockeye salmon in Chignik Lake. With only three years of observation our inferences about what causes variation in the contribution of Black Lake fish to the juvenile population in Chignik Lake are limited. We found no relationship between the proportion of Black Lake juvenile sockeye in Chignik Lake and either Black Lake temperature or the ratio of Black Lake to Chignik Lake adult spawners in the previous year. One hypothesis is that in warm years Black Lake is more stressful [59], which increases the downstream emigration rate. Similarly, greater competition during years of high densities in Black Lake could lead to increased emigration downstream. Temperature variation was very low between our study years and 2008, as was the adult escapement in the preceding years, however. In Chignik Lake, newly emerged fry are particularly susceptible to predation by coho salmon [60] and variation in predation pressure among years could alter late season stock composition in Chignik Lake. Furthermore, while sockeye dominate the pelagic fish community in Chignik Lake, the community composition has become less sockeye dominant in recent decades [61] and this could alter interspecific interactions and the opportunities for growth by Chignik Lake populations. Given the two year duration of freshwater rearing for Chignik Lake stocks, changes in predation or competition may affect the age composition and stock composition in Chignik Lake in subsequent years.

Our ability to make inferences about the attributes of a stock group depends on the success of our individual assignment. While we successfully assigned 78–80% of the individuals in our sample at an 80% probability threshold, there may be some underlying bias in the subsequent analyses based upon the individuals we were able to assign. A review of our known mixture error rate tests, however, showed that there were not differences among populations in the likelihood of not being assigned at the 80% probability threshold.

Additionally, we must be cautious when comparing mixture allocations generated using different numbers of genetic markers. In this case, differences among the proportion of Black Lake individuals observed in 2008 using 45 SNP markers and the proportions observed in 2010 and 2011 using 96 SNP markers may not be directly comparable. Instead, differences may be exacerbated or dampened by different levels of stock group differentiation between marker sets as well as the different genotypes that may be present in the samples among years.

The identification of individuals to their population of origin is essential to our ability to assess the role of migration and habitat connectivity across multiple scales of ecological organization. Emerging genetic tools offer a robust approach for investigating the presence and attributes of multiple populations within a meta-ecosystem. For species or regions where tagging studies face many logistical challenges, genetic markers provide an alternative approach that is relatively economical and efficient at tracking the stock identities of mixed-stock populations.

The consequences of movement and emigration for individuals, populations, and ecosystems can be profound. Moving to new habitat may improve growth rates over similar sized individuals [24] or allow inferior competitors the opportunity to improve growth rates [18]. Assessing the effects of alternative movement strategies on individual condition is a first step to evaluating the fitness consequences of these strategies. As habitats vary in their productivity among years, rates of migration among habitats may vary as well as the contribution of migratory individuals to population productivity [18], [19]. Migration or movement at one life stage may also alter the probability of later life history outcomes. For example, Hamann and Kennedy [62] found that juvenile Chinook salmon dispersal was related to the probability that adults would spawn in non-natal habitats. At the population level, this could affect the relative differences among populations and their fitness as well as the size of the reproductive population. Ultimately, the movement of individuals among connected habitats may drive the function and properties of meta-ecosystems by influencing trophic pathways [19] or the flux of materials among systems and in turn creating a feedback to the success of individuals and populations.

Our results highlight the importance of connectivity among the habitats that comprise a meta-ecosystem for juvenile salmonids. In the Chignik watershed, it has become apparent that Black Lake, while a more productive habitat than downstream Chignik Lake, can become unfavorable for juvenile sockeye salmon as the growing season progresses [59], [63]; this effect is particularly pronounced when lake temperatures are warmer than average [29]. Biologically compromised individuals tend to be the ones that emigrate from Black Lake [29]. Through the application of modern genetics tools, this research showed that lower performance by Black Lake emigrants continues even after moving into new habitat. The development of landscape genetics [64] has mostly focused on how the physical dimensions of landscapes affects microevolutionary processes. However, this study is one example where landscape genetics shed new perspectives on ecological processes such as migration and condition of migrating individuals. Only by understanding how individuals respond to diverse landscapes can we scale up to understanding the relative importance of different configurations of habitat networks to populations and ecosystems. Combining landscape genetics with meta-ecosystem perspectives will likely be a powerful approach for developing effective strategies for protecting and restoring habitats and their connectivity. It is becoming increasingly recognized that the connectivity of diverse habitats is important for maintaining resilient populations and the variety of ecosystem services and products they provide to people.

## Supporting Information

Figure S1 **Black Lake resident versus Chignik Lake resident body condition using alternative individual assignment probability thresholds.** Analyses were conducted using individuals assigned to stock of origin at both the 70% and 90% assignment probability thresholds. Data presented as in Figure 3.
(TIF)

Figure S2 **Black Lake resident versus Black Lake emigrant body condition using alternative individual assignment probability thresholds.** Analyses were conducted using individuals assigned to stock of origin at both the 70% and 90% assignment probability thresholds. Data presented as in Figure 4.
(TIF)

Figure S3 **Black Lake emigrant versus Chignik Lake resident body condition using alternative individual assignment probability thresholds.** Analyses were conducted using individuals assigned to stock of origin at both the 70% and 90% assignment probability thresholds. Data presented as in Figure 5.
(TIF)

Table S1 **Comparison of alternative condition factor models based upon individuals assigned at alternative probability thresholds.** Models include: the same intercept and slope for each factor (B_0_, B_1_); different intercepts and the same slope by factor (B_01_& B_02_, B_1_); same intercept but different slopes by factor (B_0_; B_11_ & B_12_); and a different intercept and slope by factor (B_01_ & B_02_; B_11_ & B_12_). AICc model weights (*w_i_*) for each threshold and year are shown for each factor comparison.
(DOCX)
