# Supplementary figures and images for: How Stock of Origin Affects Performance of Individuals across a Meta-Ecosystem: An Example from Sockeye Salmon
Source: PLoS One. 2013 Mar 7;8(3):e58584. doi: 10.1371/journal.pone.0058584 (PMC3591378; doi:10.1371/journal.pone.0058584)

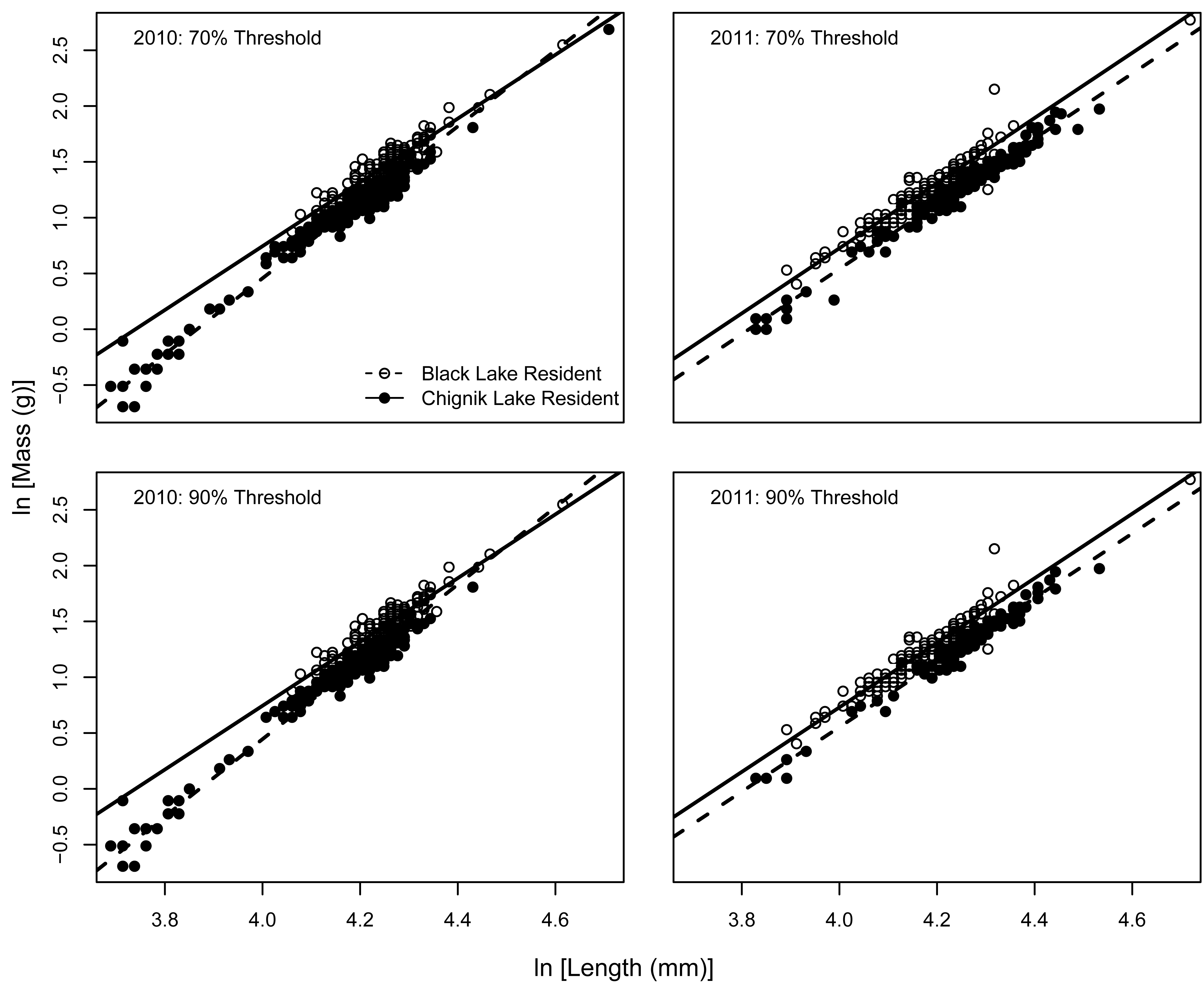

Supplement: Figure S1 — Black Lake resident versus Chignik Lake resident body condition using alternative individual assignment probability thresholds. Analyses were conducted using individuals assigned to stock of origin at both the 70% and 90% assignment probability thresholds. Data presented as in Figure 3. (TIF) [file pone.0058584.s001.tif]

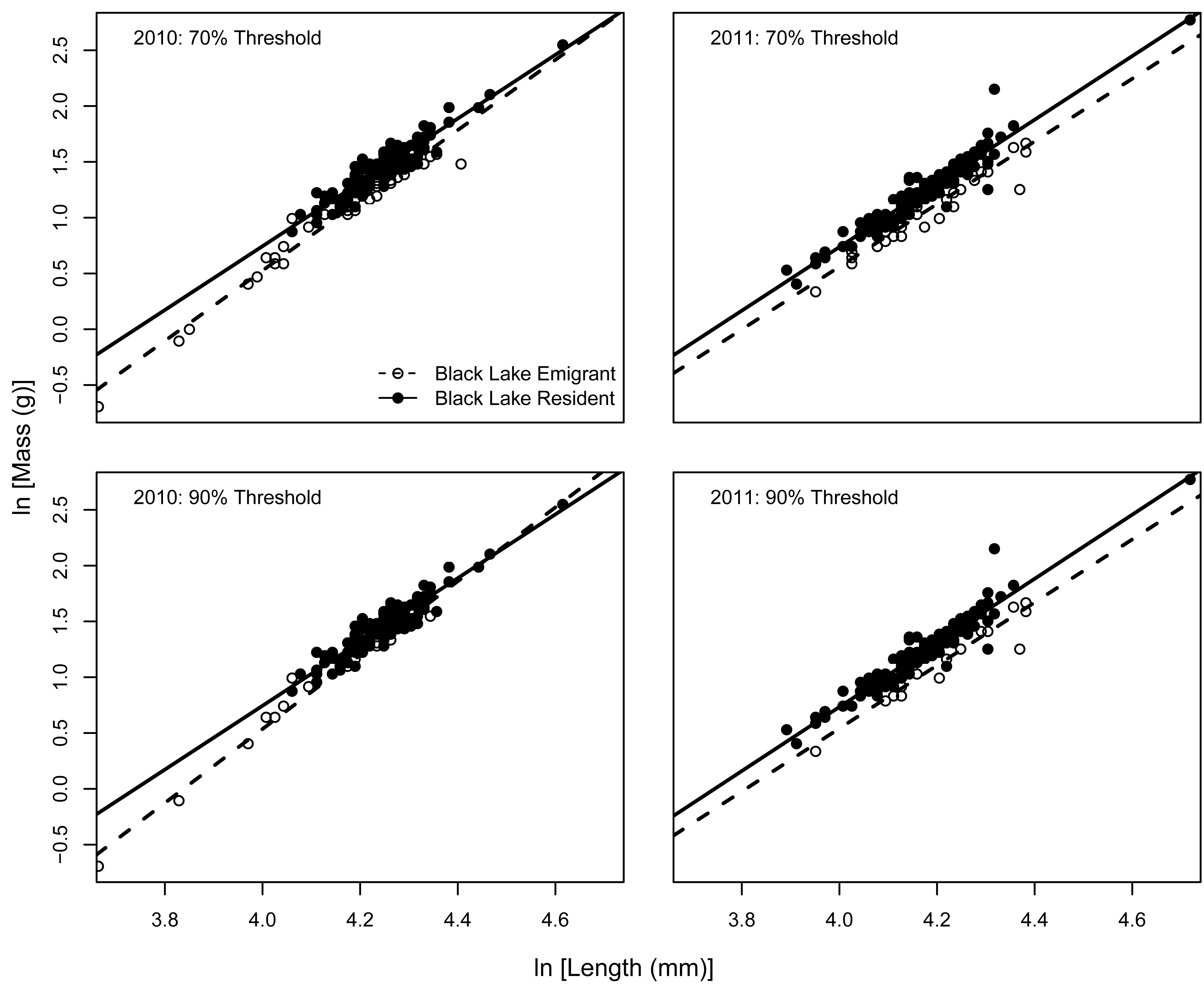

Supplement: Figure S2 — Black Lake resident versus Black Lake emigrant body condition using alternative individual assignment probability thresholds. Analyses were conducted using individuals assigned to stock of origin at both the 70% and 90% assignment probability thresholds. Data presented as in Figure 4. (TIF) [file pone.0058584.s002.tif]

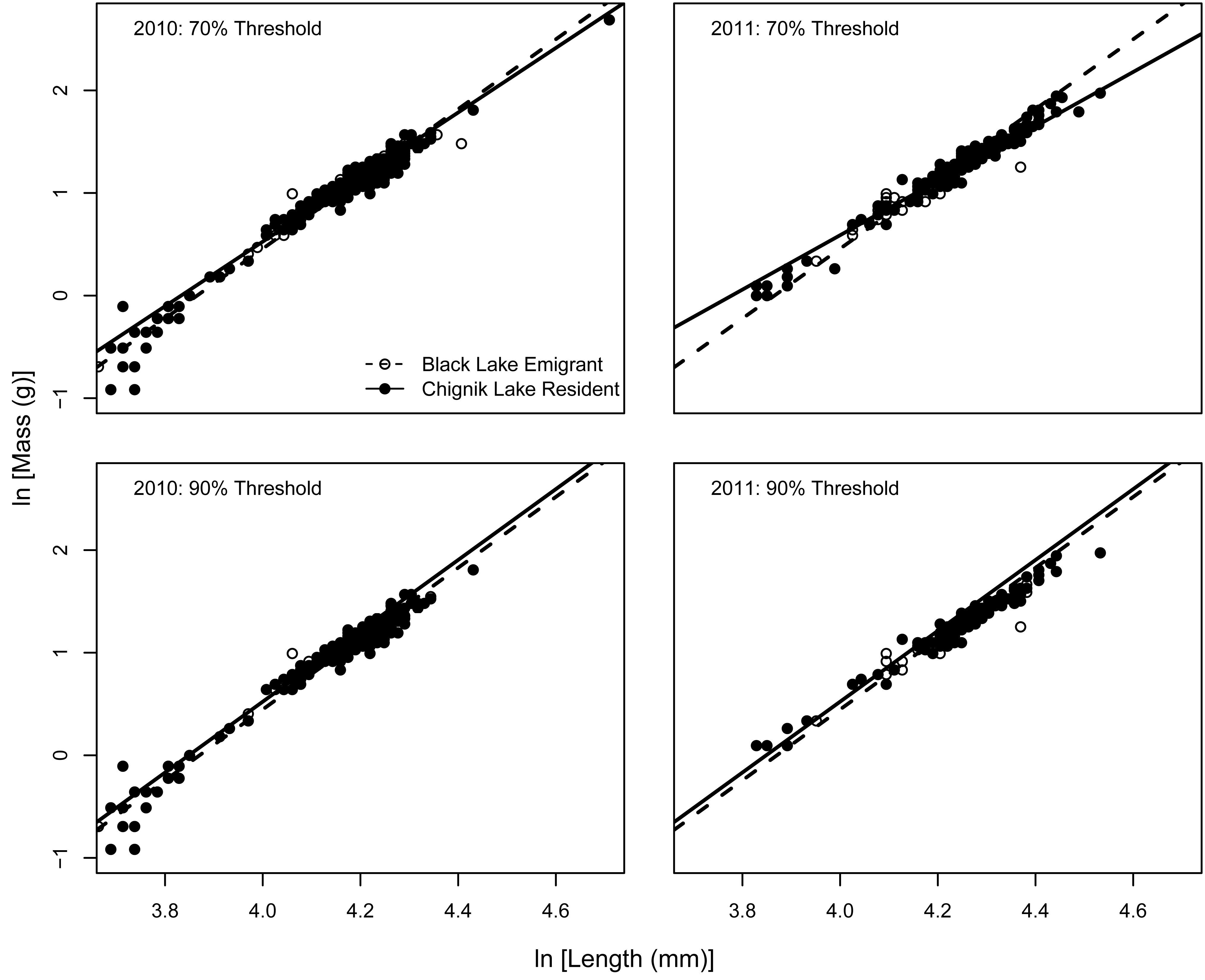

Supplement: Figure S3 — Black Lake emigrant versus Chignik Lake resident body condition using alternative individual assignment probability thresholds. Analyses were conducted using individuals assigned to stock of origin at both the 70% and 90% assignment probability thresholds. Data presented as in Figure 5. (TIF) [file pone.0058584.s003.tif]
