# Supplementary material for: How Stock of Origin Affects Performance of Individuals across a Meta-Ecosystem: An Example from Sockeye Salmon
Source: PLoS One. 2013 Mar 7;8(3):e58584. doi: 10.1371/journal.pone.0058584 (PMC3591378; doi:10.1371/journal.pone.0058584)
Supplement: Table S1 — Comparison of alternative condition factor models based upon individuals assigned at alternative probability thresholds. Models include: the same intercept and slope for each factor (B0, B1); different intercepts and the same slope by factor (B01& B02, B1); same intercept but different slopes by factor (B0; B11 & B12); and a different intercept and slope by factor (B01 & B02; B11 & B12). AICc model weights (wi) for each threshold and year are shown for each factor comparison. (DOCX) [file pone.0058584.s004.docx]

**Table S1.**.

| **Model** | **2010 w_i_** | | **2011 w_i_** | |
| --- | --- | --- | --- | --- |
|  | **70% threshold** | **90% threshold** | **70% threshold** | **90% threshold** |
| **Black L. residents & Chignik L. residents** |  |  |  |  |
| B_0_;B_1_ | 0.00 | 0.00 | 0.00 | 0.00 |
| B_01_ & B_02_; B_1_ | 0.00 | 0.00 | 0.48 | 0.43 |
| B_0_; B_11_ & B_12_ | 0.00 | 0.00 | 0.33 | 0.41 |
| B_01_ & B_02_; B_11_ & B_12_ | 1.00 | 1.00 | 0.19 | 0.15 |
| **Black L. residents & Black L. emigrants** |  |  |  |  |
| B_0_;B_1_ | 0.00 | 0.00 | 0.00 | 0.00 |
| B_01_ & B_02_; B_1_ | 0.13 | 0.02 | 0.26 | 0.33 |
| B_0_; B_11_ & B_12_ | 0.06 | 0.01 | 0.40 | 0.44 |
| B_01_ & B_02_; B_11_ & B_12_ | 0.82 | 0.97 | 0.34 | 0.23 |
| **Black L. emigrants & Chignik L. residents** |  |  |  |  |
| B_0_;B_1_ | 0.02 | 0.01 | 0.29 | 0.24 |
| B_01_ & B_02_; B_1_ | 0.06 | 0.40 | 0.10 | 0.26 |
| B_0_; B_11_ & B_12_ | 0.05 | 0.34 | 0.10 | 0.27 |
| B_01_ & B_02_; B_11_ & B_12_ | 0.87 | 0.25 | 0.50 | 0.22 |
